# Intraclass Clustering-Based CNN Approach for Detection of Malignant Melanoma

**DOI:** 10.3390/s23020926

**Published:** 2023-01-13

**Authors:** Adrian D. Bandy, Yannis Spyridis, Barbara Villarini, Vasileios Argyriou

**Affiliations:** 1 Department of Networks and Digital Media, Kingston University, London KT1 1LQ, UK; 2 Department of Electronic and Electrical Engineering, The University of Sheffield, Sheffield S1 3JD, UK; 3 School of Computer Science and Engineering, University of Westminster, London W1B 2HW, UK

**Keywords:** classification, skin lesion clustering, malignant melanoma, machine learning, CNN, medical image processing

## Abstract

This paper describes the process of developing a classification model for the effective detection of malignant melanoma, an aggressive type of cancer in skin lesions. Primary focus is given on fine-tuning and improving a state-of-the-art convolutional neural network (CNN) to obtain the optimal ROC-AUC score. The study investigates a variety of artificial intelligence (AI) clustering techniques to train the developed models on a combined dataset of images across data from the 2019 and 2020 IIM-ISIC Melanoma Classification Challenges. The models were evaluated using varying cross-fold validations, with the highest ROC-AUC reaching a score of 99.48%.

## 1. Introduction

Cancer is considered as one of the most alarming diseases nowadays, killing nearly 10 M persons each year according to the World Health Organization. Although it may come in many forms [1], skin cancer, in particular malignant melanoma, is an aggressive type that has recently seen a significant rise in the incidence rate. Melanomas can be treated with an approximately 92% survival rate if diagnosed promptly [2]. However, if the cancer is diagnosed during the later stages, this rate may drop to as low as 8%. The main factors that affect the rise of melanoma occurrences are the global aging population and increased exposure to UV radiation [3]. According to studies, a higher risk of skin cancer is observed in light-skinned people, due to low protection provided by the pigment of the higher skin layers [4].

Despite the decisive role of prompt detection, early stage diagnosis of melanoma is an arduous task due to its similarities with common moles or benign skin tumors. Neglect of such skin lesions may lead to deep invasion and wide spread of melanoma cells through the lymphatic or circulatory system. Typically, skin cancer is verified through a biopsy, using extracted samples of the suspicious skin lesion for lab analysis in a demanding process that is often time-consuming. Therefore, providing automated assistance to healthcare professionals, or supporting self-diagnosis directly to patients is of critical importance towards the early detection of melanoma. Artificial intelligence (AI), in particular machine learning (ML) or deep learning (DL) algorithms, can facilitate the development of such automated decision-support systems to enable non-invasive diagnosis procedures.

Machine learning techniques are widely used to detect meaningful patterns in varying types of data representations. Utilising specific feature selection approaches and transfer learning methods, ML algorithms have been successfully used in several medical imaging applications [5]. Due to the non-invasive nature of visual diagnoses, such techniques can be vital during early stages of diagnosis in determining the likelihood of melanomas in pigmented skin lesions [6]. More specifically, healthcare professionals can make use of diagnostic tools that employ AI models to interpret medical images and keep track of changes in skin lesions over time, thus leading to early and successful treatment of melanomas, which also implies improved cost-effectiveness in terms of medical expenses [7]. Such AI systems have been shown to offer similar accuracy in detecting skin cancer as human professionals [8].

Research in the domain of skin lesion classification through AI is broad and includes algorithms such as support vector machine (SVM) [9], decision tree [10], and k-nearest neighbor (KNN) [11]. The use of deep learning architectures such as convolutional neural networks [12] (CNNs) have proven to be the most effective at detecting and classifying skin lesions, even surpassing the abilities of expert dermatologists on some occasions [13,14].

This paper focuses in dermatoscopy image analysis (DIA) through state-of-the-art CNNs and aims to improve the results of the winning solution in the (2020) IIM-ISIC Melanoma Classification Challenge [15], in terms of ROC-AUC. Towards this goal, the base model used in [15] was retrained and fine-tuned using a combined dataset of images across data from the 2019 and 2020 competitions. Instead of focusing on binary classification, the conducted evaluation includes classification results in a varying number of classes, with primary focus given in the identification of the malignant melanoma class. Different clustering methods are investigated in a comparative analysis after performing dimensionality reduction to retain the most relevant information and improve the clustering result. Evaluation experiments indicate a small increase in ROC-AUC, with the most effective model achieving a score of 0.9948.

The rest of this paper is organized as follows. Section 2 discusses the related works conducted in the field of AI classification of skin cancer. Section 3 presents the methodology that was followed, including information about the dataset, preprocessing, the models, and the performed clusterisations. The evaluation experiments are analysed in Section 4, while Section 5 concludes this work.

## 2. Related Literature

### 2.1. Convolutional Neural Networks

Convolutional neural networks are specialised deep architectures that target visual data analysis. Typically, CNNs are characterised by the number of filters applied to the input image, where a higher number allows more subtle details and features extracted from the input. Another characteristic of their architecture is the number of layers, which affects how the CNN will be able to fit to the corresponding training data. CNNs demonstrate increased efficiency in image classification tasks due to their ability to automate the learning of useful features, as opposed to requiring human-engineered filters in other approaches.

The study in [16] demonstrates the use of a deep CNN to perform binary classification of malignant melanoma or benign nevus in proven clinical images. The authors use the Google’s Inception v3 architecture to train their model on a big dataset including 129,450 cases. The results were compared with a group of certified dermatologists, demonstrating that the AI is capable of performing similarly to trained experts.

An ensemble of CNN models was employed to efficiently identify melanomas using skin images along with patient metadata in [15], providing the winning solution to the 2020 IIM-ISIC Melanoma Classification Challenge by achieving a classification accuracy of 98.45%. The authors performed a fivefold cross validation on the combined data provided by the competition during the last three years, achieving a more stable performance across all possible classification thresholds, as shown by the AUC metric. Their method employed the popular EfficientNet, which is a pretrained CNN model for general purpose computer vision tasks, and fine-tuned it on the provided dataset by changing the structure of the last layer.

### 2.2. Support Vector Machines

SVMs belong to the class of supervised machine learning algorithms that specialise in data analysis for classification problems. Based on the concept of hyperplanes, SVMs target to select the support vector, which is the hyperplane that maximises the distance to the nearest data point of the related classes. SVMs perform well in linear classification tasks, but are also suitable for non-linear data by mapping the inputs to high-dimensional spaces through various kernel functions.

A nonlinear SVM classifier was used to detect melanomas of affected skin lesions in [17] by extracting features from preprocessed images, combined with a mean shift segmentation algorithm. Using 200 images, divided into 70% training and 30% testing data, experiments demonstrated that 6 out of the 15 extracted features are adequate to provide a consistent performance. The classification model was able to achieve an 86.67% accuracy, with an F1-score of 0.9091. Similarly, in [18], the study employed an SVM classifier to identify the presence of melanoma in dermatoscopic images. The feature extraction involved statistical texture and color features on the affected lesions in varying structures. First, a 13-dimensional vector was constructed based on 9 color and 4 texture characteristics, and then the SVM model was used to classify given melanoma images. The classifier was evaluated on the PH2 dataset and achieved an accuracy of 96% when using the combined features.

In [19] the type of skin cancer was detected using an SVM classification method by extracting histogram of gradient (HOG) features in dermatoscopic images. These features were mapped to seven class labels using the radial basis function (RBF) kernel. The classification model was evaluated on the ISIC 2018 dataset, achieving a 76% accuracy with an F1-score of 75%. The SVM algorithm was enhanced in [20] through a heuristic optimisation algorithm to develop a hybrid classification method named hybSVM. The new algorithm targets optimising the gamma and C value to improve the operating cost. This method was evaluated using both the ISIC 2018 dataset, and the PH2 dataset, demonstrating a high time efficiency, while achieving at least 97.5% accuracy in each dataset.

### 2.3. Decision Trees

In the context of machine learning, decision trees are supervised learning algorithms that primarily target classification problems. These classifiers utilise a tree structure, whereby each node represents a feature of the dataset. Leaf nodes are used to represent the class labels, and branches represent the decision conditions. Typically, such decision trees are built using the CART algorithm.

A skin cancer detection system based on a decision tree classifier is presented in [21]. Input images are preprocessed to remove hair artifacts in the affected lesions through the dull-razor technique, which are then isolated using the active contour segmentation method. Consequently, the system extracts color features after converting the image to HSV and YCbCr color spaces. Following the evaluation of the system’s performance in the MED-NODE dataset using different classification algorithms, the decision tree is found to outperform naive Bayes and KNN classifiers, achieving an 82.35% accuracy.

Using the ID3 algorithm, a decision tree is constructed in [22] to classify dermatoscopic images into one of four classes, including healthy, melanoma, eczema, and leprosy. The images are preprocessed through grayscale conversion together with contrast enhancement and are then segmented using the global value thresholding method. The detection system extracts features such as energy, entropy, contrast, IDM, correlation, and ASM, achieving a classification accuracy of 87%. Similar texture features are extracted in [23], based on a hybrid discrete wave transform and principal component analysis approach. These features fed a naive Bayes and a decision tree classifier to detect malignant melanomas in images from the DIS and DermQuest datasets. The naive Bayes was able to outperform the decision tree, achieving an accuracy of 98.8%, compared to 92.86%.

While several methods presented in the literature have proven their effectiveness in melanoma classification, automated detection systems built around them still face limitations due to limited data availability that often causes racial bias with respect to certain skin types. This limited data availability hinders the ability to further improve current state-of-the-art models. These limitations are expected to be resolved when technologies such as infrared, optical coherence tomography, and confocal microscopy [24] are utilised to develop new data. Besides these limitations, the study presented in this paper offers the advantage of automatically selecting features used during the model training, with techniques such as dimensionality reduction and AI clustering, instead of requiring manual selection by experts.

## 3. Methodology

### 3.1. Dataset

The dataset employed in this work includes images from both the ISIC 2020 and the 2019 melanoma classification datasets. After the removal of duplicate records, the combined dataset includes 48,007 images from approximately 2000 patients of all age groups. Each record belongs to one of nine classes, as outlined in Table 1. The dataset includes a column with “tfrecord” values, allowing evaluation with up to 15 k-fold validation. These values were stratified so that the melanoma proportion for each record is balanced to contain the same percentage, along with a similar distribution of images.

### 3.2. Base Model

**Figure 1:**
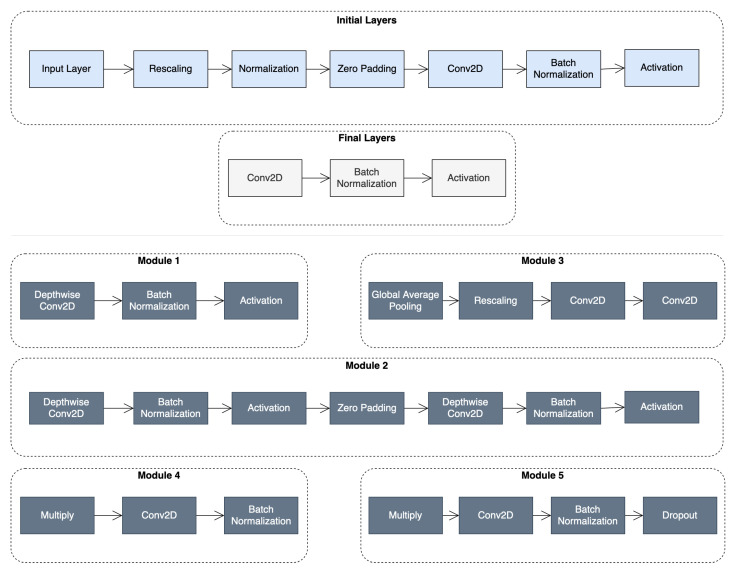
**Top**: the initial and final layers in the baseline EfficientNet architecture; **Bottom**: the types of modules that comprise the architecture.

The base model uses the EfficientNet-B6 architecture, originally pretrained on ImageNet and then trained on the employed dataset, due to its exceptional performance in transfer learning tasks, while keeping the number of trained parameters significantly low. The architecture comprises 667 layers built from the modules shown in Figure 1, reaching a total number of trainable parameters of approximately 41 M.

The loss function utilised for the training is cross entropy loss, given by Equation (1):

(1) $$L=-\sum_{k=1}^{n}t_{k}log\left(p_{k}\right),$$

where $t_{k}$ denotes the truth label, and $p_{k}$ denotes the probability of the $k^{th}$ class. The Softmax probability is given by Equation (2):

(2) $$\sigma {\left(z\right)}_{i}=\frac{e^{z_{i}}}{\sum_{j=1}^{K}e^{z_{j}}},$$

where $z_{j}$ are the elements of the output vector. The models are trained through gradient descent using the Adam optimiser.

### 3.3. Clustering Overview

The primary focus of this work lies in the identification of melanoma in the provided dataset. This gives rise to the possibility of grouping the rest of the data in different ways to create various clustering categories in an effort to increase the melanoma classification performance. To obtain useful clustering information, the images were passed through the base model, retrieving the values of the penultimate layer which includes 2304 features. Several clustering techniques were employed, as outlined in Section 3.5, Section 3.6, Section 3.7 and Section 3.8, and evaluated in a comparative analysis to determine which will yield the optimal performance with the base model. Before clustering, all features were rescaled using unity-based normalisation.

### 3.4. Dimensionality Reduction

**Figure 2:**
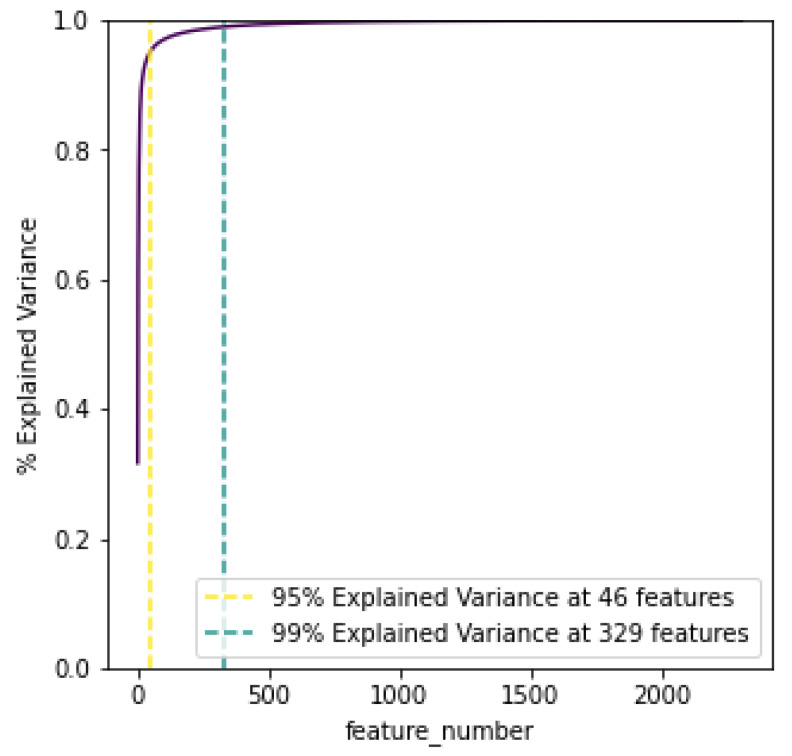
PCA cumulative explained variance.

Dimensionality reduction was performed on the data using principal component analysis (PCA), to reduce the amount of the 2304 retrieved features. The aim of using PCA was to capture the components that describe the largest explanations of variation and list them in order of importance. As showcased later in the evaluation, this process improves the time efficiency of the clusterisation, but also results in higher performance metrics. Overall, PCA helps in alleviating the effect of the “Curse of Dimensionality”. By creating a cumulative total of the explained variance, the graph in Figure 2 plots how many PCA features are needed for 95% and 99% variance.

### 3.5. Medical Prognosis Based Clustering

The primary focus of this work is to improve the melanoma classification rate through AI clustering methods. However, it was deemed necessary to examine a non-algorithmic clustering approach as well to obtain useful insight about the potential categories. This clusterisation was designed based on the relationship between each class and the priority of seeking medical expertise, as described in [25]. The obtained classes were categorised according to suspicious lesions, which require immediate medical referral, non-suspicious lesions B, and non-suspicious lesions A, which require medium and low priority, respectively.

### 3.6. K-Means

**Figure 3:**
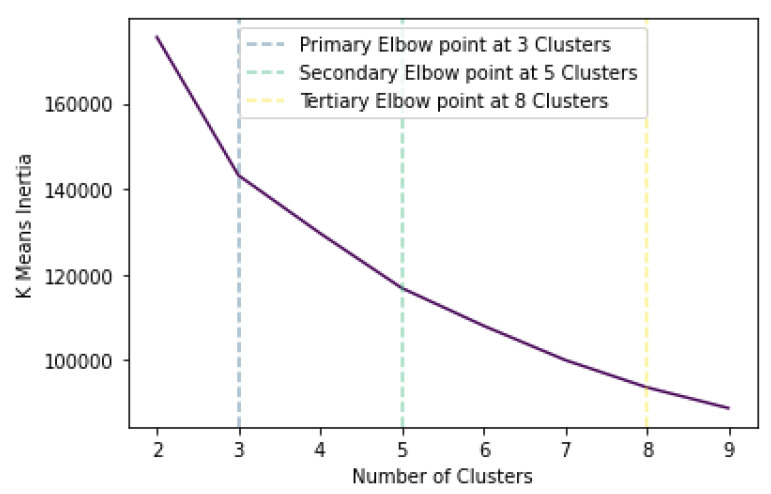
K-means inertia versus number of clusters.

K-means clustering aims to categorise *m* points inside *n* dimensions into *k* clusters so that the sum of squares in each cluster is minimised. In essence, through k-means the points are categorised based on their proximity in the Euclidean space. To determine the suitable number of clusters, the measure of within-cluster sum of squares, called inertia, is calculated and plotted for a varying number, as demonstrated in Figure 3. As observed, the inertia decreases as the number of clusters increases, which is expected since as the number of clusters approaches the number of points, the distance is minimised. Figure 3 indicates 3 as the primary elbow point, which suggests this as the number of optimal clusters. This can also be verified by calculating the silhouette score, which aims to describe how well each label fits into its designated cluster. It should be noted that the optimal formation of clusters does not necessarily mean that melanoma would be distinctly separated in its own cluster. This was determined by calculating precision and recall scores in each case to obtain the optimal clustering groups.

### 3.7. Hierarchical Clustering

Two methods of hierarchical clustering were utilised: single-linkage and complete-linkage. The first performs clustering based upon the minimum distance between any point in that cluster and the data point being examined. In contrast, complete linkage performs clustering based upon the minimisation of the maximum distance between any point in the cluster and the data point being examined. Hierarchical clustering may lead to unequal cluster sizes, but since the classes in the employed dataset are not balanced, this does not affect this work.

**Figure 4:**
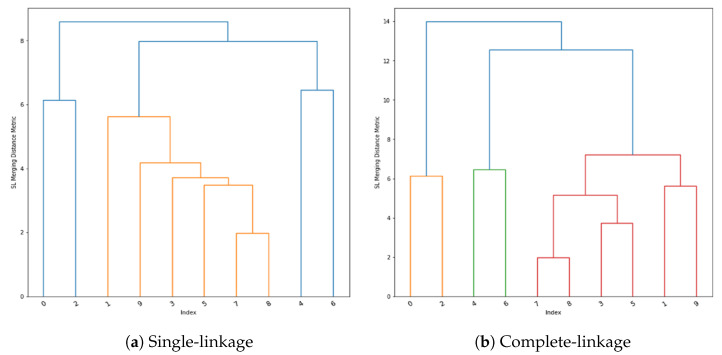
Hierarchical clustering dendrograms.

The approach of single-linkage is demonstrated as a dendrogram in Figure 4a. The data points are observed at indexes between 0 and 9, with higher lines between 2 indexes indicating how further apart they are in terms of the linkage metric. The dendrogram in Figure 4b demonstrates complete-linkage clustering.

### 3.8. TSNE Clustering

**Figure 5:**
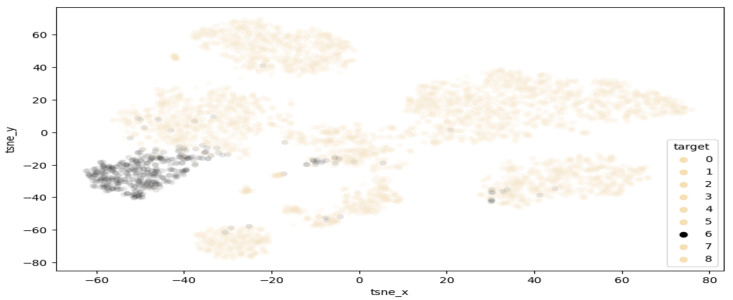
Highlighted separation of melanoma from other classes.

**Figure 6:**
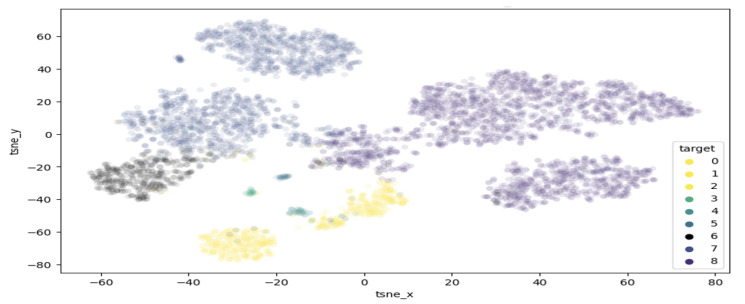
Proposed clusterisation through TSNE.

T-distributed stochastic neighbour embedding (TSNE) is a method of visualising multi-dimensional space in two dimensions. This approach is of high interest in this work, as it can help highlight clusters of samples within the melanoma class. Figure 5 demonstrates the results of performing TSNE clustering on PCA-reduced data, highlighting the range of values falling into the melanoma class. While a clearly visible grouping exist around −50 $tsne_{x}$ and −30 $tsne_{y}$, there are also a few points outside the main cluster, which are considered extreme values and may contain features unlike most melanomas. Figure 6 displays the proposed clustering through TSNE. As observed, classes 0–4 were grouped together, while each other class comprises a unique cluster.

The TSNE approach was also examined on the PCA data with 95% explained variance, utilising the top 46 PCA features. Finally, the k-means clustering was also applied to the TSNE data after reducing them with PCA and after using the 95% explained variance features as well.

## 4. Results

To validate the performance of the study, evaluation metrics were calculated on the experiments conducted in the training and classification steps, using a subset of 5024 images taken from the combined dataset.

### 4.1. Clustering Categories

**Figure 7:**
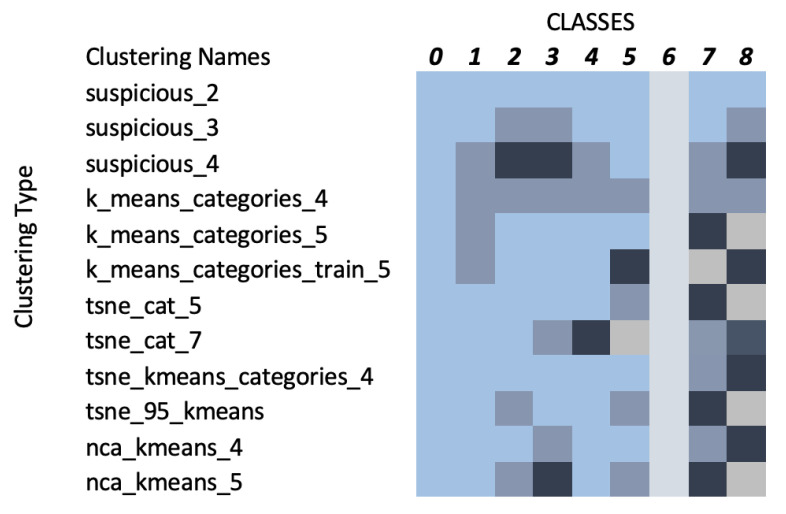
Relationship between clusters and target classes (each color represents a different cluster).

Following the dimensionality reduction using PCA, the clustering study proposed a variety of class groupings, as illustrated in Figure 7. Each color inside a row represents a different cluster. Suspicious_2, 3, and 4 denote the clustering as described in Section 3.5. K_means_categories_4, 5, and train_5 denote the clustering as described in Section 3.6, while tsne_cat_5 and 7 and tsne_kmeans_categories_4 denote the clustering as presented in Section 3.8. Finally, tsne_95_kmeans denotes the clustering based on Section 3.8, while using PCA 95% variance data and nca_kmeans_4 and nca_kmeans_5 denote the clustering according to neighbourhood component analysis (NCA). The NCA method utilised a distance metric based upon standard deviation and nearest neighbours, performing a linear transformation on the data which produced some unique clustering results compared to the other methods since in NCA each point inherits a class label from its neighbour based on a certain probability to select that neighbour point [26]. Based on the focus of this study, all proposed schemes resulted in a distinct Class 6, which corresponds to melanoma.

### 4.2. Performance Evaluation

The performance is assessed using the AUC and ROC-AUC metrics. The ROC curve is plotted based on the recall versus precision, where the first indicates the true positive rate, calculated as $\frac{TP}{TP+FN}$, and the second indicates the number of positive predictions that are actually positive, calculated as $\frac{TP}{TP+FP}$. True positives correspond to the values of correctly identified skin lesions, while false positives indicate the classes that were incorrectly identified as melanomas. False negatives correspond to the values of melanomas that were not correctly identified. All experimental scenarios are evaluated based on the AUC and ROC-AUC scores, as presented in Table 2 and Table 3, respectively.

#### 4.2.1. AUC Scores

To calculate the performance of the model across all classification thresholds, AUC is used to calculate the Area under the ROC curve, which effectively measures the probability that a positive sample will be ranked higher than a negative sample. This metric is selected due to its invariant nature both in terms of scaling and in terms of classification threshold. Table 2 outlines the AUC score as calculated in a variety of cases where the last layer is retrained using the methods of this paper. The Base model refers to the winning model presented in [15], trained using the dataset employed in this study.

As observed in the table, model T_22 was able to achieve the highest AUC score and therefore was retrained using different learning rates. A further increase was therefore achieved with the final score reaching 0.9962, thus a 0.1% improvement over the base model.

#### 4.2.2. QDA & LDA

**Figure 8:**
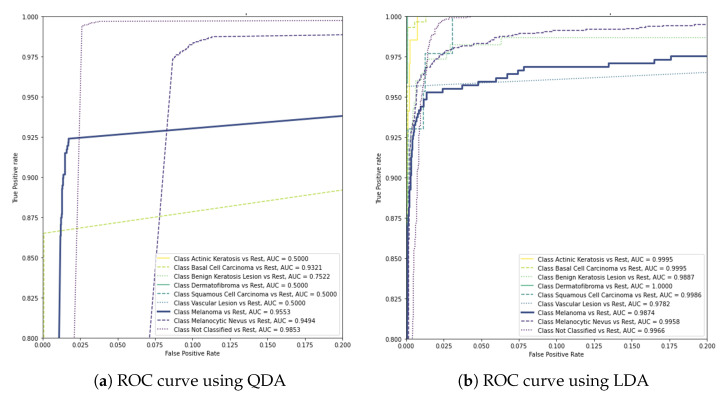
AUC scores of QDA and LDA in the final layer of the base model.

In addition to the previous experiments, the evaluation studied quadratic discriminant analysis (QDA) and linear discriminant analysis (LDA) as alternatives to the final layer of the base model to investigate a non-linear and a linear boundary between the classifiers, respectively. As demonstrated in the graphs of Figure 8, these models were not able to achieve the same performance with the previous approaches, reaching scores of 0.9553 and 0.9874, respectively.

#### 4.2.3. ROC-AUC Scores

The conclusive evaluation was conducted by performing k-fold validations, calculating the final ROC-AUC score for selected models. Each test image was evaluated in eight different transposes/flips, and the average result was obtained. The final scores are outlined in Table 3.

**Figure 9:**
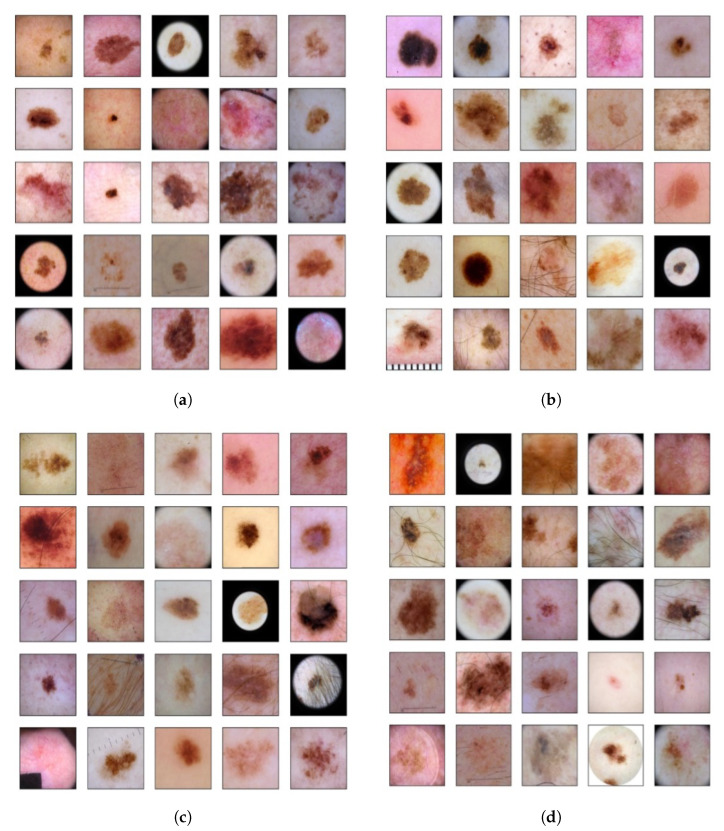
(**a**) True positives in the top model that appeared as false negatives in the base model; (**b**) true negatives in the top model that appeared as false positives in the base model; (**c**) false negatives in the top model that appeared as true positives in the base model; (**d**) false positives in the top model that appeared as true negatives in the base model.

The top models were able to slightly outperform the base model, achieving scores of 99.48%. While further improvements to the base model were expected to yield diminishing returns. Since it is based on state-of-the-art designs and data augmentation, this study was able to achieve a baseline increase of 0.05% in the ROC-AUC score when compared to [15]. Figure 9 depicts selected cases where the top developed model was able to outperform the base model in terms of true positives (Figure 9a) and true negatives (Figure 9b), while it resulted lower number of false negatives (Figure 9c) and false positives (Figure 9d). These figures also provide insight with regard to the range of visual characteristics that malignant melanomas display and showcase the inclusion of melanoma-like traits in non-positive cases as well.

**Figure 10:**
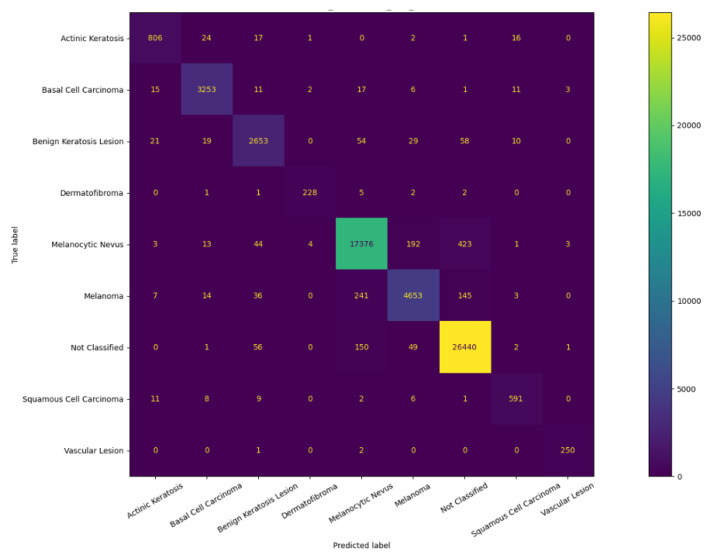
Confusion matrix of the base model.

These results are more clearly illustrated by the confusion matrix presented in Figure 10. Small improvements over the base model were also observed in terms of precision and F1-score (see Equation (3)), which further indicate the accuracy of the model as listed in Table 4. This increase in precision suggests that compared to the base model, the developed models are able to correctly identify a higher proportion of melanoma cases among all positive cases. Taking all metrics into consideration, the study concludes that model E_7 is the most effective classification model. The results also suggest that classification of the desired class may be achieved by inferring appropriate categories based on AI-assisted clustering instead of relying on the designated diagnosis of each category.

(3) $$F1-\mathrm{score}=2\times \frac{precision\times recall}{precision+recall}$$

## 5. Conclusions

As the incidence rate of skin cancer increases, non-invasive diagnosis approaches are becoming more essential for prompt detection and treatment of malignant melanomas. Towards this goal, this study developed a highly effective classification model based on a state-of-the-art CNN architecture. Based on a comparative analysis of varying clustering methods, the base neural network was retrained through the mapping of the target labels as the result of the feature output clustering. Evaluation experiments demonstrated how this approach was able to improve the performance of the winning model in the 2020 IIM-ISIC Melanoma Classification Challenge in terms of ROC-AUC, reaching a value of 99.48%. Additional improvements were observed with respect to precision and F1-score, with values of 95.19% and 92.72%, respectively.

As of the writing of this paper, the use of CNNs for medical diagnosis is generally not permitted in health institutions. This is also encouraged by current GDPR regulations which state that all patients have a right to explanation with respect to processing of their personal data, therefore conflicting with such automated diagnosis systems. Considering the above, the melanoma classification model developed in this work is not supposed to be a substitute for a dermatologist or further analytical procedures deemed necessary, rather it is designed to alert experts and provide valuable insight regarding potential cases, which can assist in prioritisation and early detection of suspicious lesions. As a result, the outcomes of this study outline the potential of developing AI-assisted decision support systems as fast and reliable tools to augment dermatologists during the diagnosis procedure.

## Figures and Tables

**Table 1:** Description of the dataset.

Class	Target	Sample Size
Melanoma	6	5099
Melanocytic Nevus	7	18,059
Basal Cell Carcinoma	1	3319
Benign Keratosis Lesion	2	2844
Actinic Keratosis	0	867
Squamous Cell Carcinoma	4	628
Vascular Lesion	5	253
Dermatofibroma	3	239
Not Classified	8	26,699

**Table 2:** AUC scores for the different models studied.

Model	Description	AUC Score
Base	Base model	0.9949
T_17	Retrained on binary classification	0.9935
T_18	Retrained on Melanoma, Suspicious, Not Suspicious	0.9945
T_19	Retrained on Melanoma, Suspicious, Not Suspicious A and B	0.9945
T_20	Retrained on the 4 classes, using PCA and k-means	0.9941
T_21	Retrained on the 4 classes, using TSNE and k-means	0.9951
T_22	Retrained on 5 classes, using PCA and k-means, lr = $3\times 10^{-5}$	0.9952
T_23	T_22, lr = $1.5\times 10^{-5}$	0.9939
T_24	T_22, lr = $1.5\times 10^{-6}$	0.9960
T_25	T_22, lr = $5.5\times 10^{-6}$	0.9962
T_26	T_22, lr = $5.5\times 10^{-7}$	0.9960
T_27	Retrained on 5 classes using TSNE directly	0.9950
T_28	Retrained on the 4 classes, using TSNE and k-means	0.9948
T_29	Retrained on 7 classes using TSNE directly	0.9951

**Table 3:** ROC-ACU scores for the different models studied.

Model	Description	ROC-AUC
Base	Base model	0.9943
E_1	Retrained on 5 classes, using PCA 95% TSNE and k-means	0.9946
E_2	Retrained on 4 classes, using NDA and k-means	0.9947
E_3	Retrained on 5 classes, using NDA and k-means	0.9945
E_4	Retrained on binary classification	0.9941
E_5	Retrained on 4 classes, using TSNE k-means	0.9946
E_6	Retrained on 5 classes, using TSNE k-means	0.9947
E_7	Retrained on 5 classes, using PCA and k-means	0.9948
E_8	Retrained on 5 classes, using TSNE	0.9946
E_9	Retrained on 7 classes, using TSNE	0.9948

**Table 4:** Precision, recall, and F1-score of the top models.

Model	Description	ROC-AUC	Precision	Recall	F1 Score
Base	Base model	0.9943	0.9421	0.9125	0.9271
E_7	Retrained on 5 classes, using PCA and k-means	0.9948	0.9519	0.9076	0.9292
E_9	Retrained on 7 classes, using TSNE	0.9948	0.9448	0.9102	0.9272
